# Micro-Economics of Apoptosis in Cancer: ncRNAs Modulation of BCL-2 Family Members

**DOI:** 10.3390/ijms19040958

**Published:** 2018-03-23

**Authors:** Lidia Villanova, Silvia Careccia, Ruggero De Maria, Micol E. Fiori

**Affiliations:** 1 Department of Oncology and Molecular Medicine, Istituto Superiore di Sanità, 00161 Rome, Italy; lidia.villanova@gmail.com; 2 Institute of General Pathology, Catholic University of the Sacred Heart and Gemelli Polyclinic, 00168 Rome, Italy; silvia.care@gmail.com

**Keywords:** microRNAs, long non-coding RNAs, BCL-2 family, apoptosis, cancer, therapy resistance

## Abstract

In the last few years, non-coding RNAs (ncRNAs) have been a hot topic in cancer research. Many ncRNAs were found to regulate the apoptotic process and to play a role in tumor cell resistance to treatment. The apoptotic program is on the frontline as self-defense from cancer onset, and evasion of apoptosis has been classified as one of the hallmarks of cancer responsible for therapy failure. The B-cell lymphoma 2 (BCL-2) family members are key players in the regulation of apoptosis and mediate the activation of the mitochondrial death machinery in response to radiation, chemotherapeutic agents and many targeted therapeutics. The balance between the pro-survival and the pro-apoptotic BCL-2 proteins is strictly controlled by ncRNAs. Here, we highlight the most common mechanisms exerted by microRNAs, long non-coding RNAs and circular RNAs on the main mediators of the intrinsic apoptotic cascade with particular focus on their significance in cancer biology.

## 1. Intrinsic Apoptotic Program

The word “apoptosis” was coined from Greek to address a programmed cell death, which contributes to the tissue homeostasis as the “falling off” of autumn leaves in a tree [1]. The apoptotic machinery consists of both upstream regulators and downstream effectors [2]. The regulators are divided into two major circuits, one processing extracellular death-inducing cues (the “extrinsic” apoptotic program, involving for example the Fas ligand/Fas receptor) and the other (the “intrinsic” or “mitochondrial” program) sensing a variety of stress signals of intracellular origin such as DNA damage, accumulation of reactive oxygen species (ROS), Ca^2+^ overload, survival factors’ deprivation or oncogene activation. Each program leads to the activation of a normally latent cysteine protease (caspase 8 or 9, respectively), which triggers a cascade of proteolytic events involving effector caspases 3 and 7 responsible for the execution phase of apoptosis, resulting in the progressive disassembly of cellular structures and ultimately engulfment by phagocytic cells.

Mitochondria are the cells’ energy factories, but also the organelles where suicidal signals converge and are executed. The intrinsic apoptotic program is more widely implicated as a barrier to cancer pathogenesis [3]. It culminates in mitochondrial outer membrane permeabilization (MOMP), followed by the release into the cytosol of cytotoxic proteins normally confined within the mitochondrial intermembrane space (IMS). Such cytotoxic proteins include caspase activators such as cytochrome C and DIABLO, as well as caspase-independent cell death effectors like apoptosis-inducing factor and endonuclease G.

Proteins belonging to the BCL-2 family are the key regulators of MOMP. This family consists of three groups of structurally-related proteins: the pro-survival BCL-2-like proteins (BCL-2, BCL-XL, BCL-W, MCL-1, BFL-1), the pro-apoptotic multi-BH domain BAX/BAK proteins and the pro-apoptotic BH3-only proteins (BIM, PUMA, BID, BAD, BIK, BMF, NOXA, HRK). In healthy cells, BAX and BAK are kept inactive by the interaction with pro-survival members [4,5]. In response to pro-apoptotic signals, BAX and BAK can trigger MOMP by inducing the formation of protein-permeable pores in the mitochondrial outer membrane that allow the release of IMS proteins into the cytosol [6]. BH3-only proteins share significant homology within the BCL-2 homology 3 (BH3) domain and act as intracellular stress sensors. All BH3-only proteins can indirectly activate BAX/BAK by sequestering their pro-survival BCL-2-like relatives, whereas only some of them can also trigger BAX/BAK activation through direct binding (Table 1) [7,8].

## 2. BCL-2 Family Members in Cancer Onset, Progression and Resistance to Therapy

Evasion of apoptosis has been classified as one of the hallmarks of cancer [9] and is among the main causes of therapy failure. Most tumors are defective in the activation of apoptosis owing to the occurrence of genetic or epigenetic events that either inactivate the p53 pathway or provoke aberrant expression of pro-survival BCL-2 family members including BCL-2, BCL-XL or MCL-1 [10,11]. It has been shown that BCL-2 exerts a key role in tumorigenesis. In a mouse model of APC-loss induced colorectal cancer, BCL-2-dependent impairment of apoptosis is required for carcinoma onset [12]. Importantly, such unbalance in favor of the pro-survival side of the BCL-2 family confers resistance to radiation, chemotherapeutic agents and many selective pathway inhibitors that induce apoptosis primarily through the activation of the mitochondrial death machinery. Notably, a recently-developed functional assay called “BH3 profiling” allows assessing the extent of mitochondrial sensitivity to the induction of MOMP by measuring mitochondrial depolarization upon cells’ exposure to peptides derived from BH3-only proteins. BH3 profiling of tumors has been proposed as a tool for predicting chemotherapy sensitivity, since the extent of “mitochondrial priming” in several tumor types was found to correlate with clinical response to cytotoxic chemotherapy [13].

Noteworthy, some BCL-2 family proteins are involved in other cancer hallmarks besides apoptosis resistance. BCL-W expression was reported to be upregulated in the infiltrative types of gastric cancer [14] and in glioblastoma multiforme biopsies (WHO Grade IV) compared with glioma (WHO Grade III) and normal tissues [15], suggesting a role in tumor progression. Bae and colleagues provided a mechanistic insight, showing that BCL-W ectopic expression enhanced the invasive potential of gastric cancer and glioblastoma cell lines by inducing MMPs expression via a PI3K-AKT-mediated activation of SP1 and β-catenin, respectively [15,16].

In a bi-transgenic mouse model engineered to spontaneously develop pancreatic neoplasia, BCL-XL viral transduction in hyperplastic or early dysplastic lesions increased tumor invasion and facilitated lymph node metastasis, without altering cancer cell survival or proliferation [17]. BCL-XL-induced increase in the migratory and invasive potential was confirmed in pancreatic cancer cell lines, and a remodeling of the actin cytoskeleton was observed in both cell cultures and pancreatic cancer xenografts. Downregulation of E-cadherin expression and co-immunoprecipitation of BCL-XL with myosin Va, besides BAX and BAD, shed light on the molecular mechanisms underlying the observed phenotypes. More recently, BCL-XL was shown to promote stemness and to contribute to the aggressiveness of both melanoma and glioblastoma [18]. Further, by testing apoptosis-defective mutants in pancreatic neuroendocrine tumor (panNET) and breast cancer cell lines, Choi and colleagues demonstrated that BCL-XL promotes metastasis independently of its anti-apoptotic activity. They provided evidence for BCL-XL nuclear functions involving epigenetic activation of TGFβ promoter, thus boosting TGFβ signaling [19].

BCL-2 was reported to have a role in tumor onset, spread and therapy resistance that does not rely uniquely on the apoptotic pathway. Human lung epithelial cells chronically exposed to the carcinogenic hexavalent chromium Cr(VI) undergo transformation that strongly relies on BCL-2. Medan and colleagues showed that BCL-2 drives invasive and proliferative properties of the transformed cells, as well as their colony-forming and angiogenic activities [20]. Further, BCL-2 was found to stimulate epithelial to mesenchymal transition (EMT) in pre-malignant mammary epithelial cells and in hepatocarcinoma cells, where the formation of a BCL-2/TWIST1 complex facilitates the nuclear transport of TWIST1, leading to transcriptional activation of TWIST target genes that sustain tumor cell plasticity, metastasis and vasculogenic processes [21,22].

Overall, these results point out the involvement of BCL-2, BCL-W and BCL-XL in molecular events not related to the apoptotic cascade. These “other” cellular activities have been linked to the BH4 domain, which mediates direct interactions with specific proteins functionally implicated in distinct stages of tumor progression. Lately, recent efforts are aiming at developing new inhibitors directed against the BH4 domain, which is considered an appealing target due to its unique structure and crucial involvement in many cellular functions [23].

## 3. Different Layers of Regulation of BCL-2 Family Members by ncRNAs

BCL-2 family proteins are subjected to several layers of regulation that affect transcript levels and protein half-life, phosphorylation status and localization. Besides p53-mediated transcriptional activation of PUMA and NOXA following DNA damage [24], other transcription factors were found to regulate BH3-only proteins including E2F1 [25], FOXO3a [26], CHOP [27] and NF-Y [28]. Interestingly, NF-YA was found to play a role in the differential kinetics of the induction of p53 target genes upon cellular stress. Indeed, orchestration of p53 stress response relies on earlier expression of cell cycle arrest-related genes compared to pro-apoptotic genes [29]. This is accomplished by NF-YA recruitment to the promoters of the pro-apoptotic subset, where it affects RNA PolII recruitment and processivity [30]. Control over protein stability is exemplified by MCL-1 degradation by the ubiquitin ligases MULE and FBW7 [31,32]. In addition to modifying protein levels, activities of BCL-2 proteins including BCL-XL, NOXA, BIM, BAK and BAX can be modified by protein phosphorylation [33,34,35,36,37,38]. Further, the localization of BCL-2 proteins can be influenced by upstream factors. For example, BAX is constrained at the Golgi until being released by DNA damage signals [39] or maintained in the cytoplasm by BCL-XL [40]. Finally, the expression of the BCL-2 family proteins can be modulated through the activity of ncRNAs.

**Figure 1:**
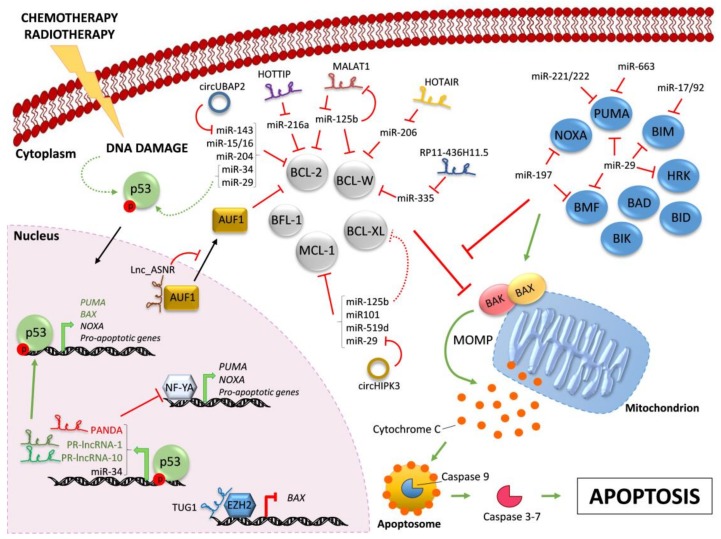
Regulation of BCL-2 family members by ncRNAs. Chemotherapy and/or radiotherapy induce DNA damage that leads to the activation of p53 (dashed curve green arrow). Active p53 translocates into the nucleus and drives the transcription (bold green arrows) of the indicated pro-apoptotic proteins and ncRNAs. Indicated lncRNAs control the transcription of pro-apoptotic proteins by different mechanisms. The pro-apoptotic multi-BH domain BAX and BAK are kept inactive by the interaction with pro-survival BCL-2-like proteins (in grey). All BH3-only proteins (in blue) can indirectly activate BAX/BAK by sequestering their pro-survival BCL-2-like relatives (red line), whereas only some of them can also trigger BAX/BAK activation through direct binding (green arrow). Active BAX/BAK oligomerize on the mitochondria membrane and lead to mitochondrial outer membrane permeabilization (MOMP) and consequent Cytochrome C release in the cytoplasm. Activation of downstream caspase cascade ultimately triggers apoptosis. In the cytoplasm, microRNAs negatively regulate BH3-only proteins and pro-survival BCL-2 family members. Indicated lncRNAs and circRNAs act as miR-sponges, counteracting miR-mediated regulation of pro-survival proteins. Further, *MALAT1* is down-regulated by miR-125b, in a negative feedback-loop. Red lines indicate inhibition; green arrows indicate activation. Continuous arrows and lines refer to direct regulation; dashed arrows and lines indicate indirect regulation. Black arrows indicate nuclear/cytoplasmic translocations.

Here, we aim to outline the most relevant regulatory strategies involving ncRNAs that human cells evolved to control cell death and their implications in cancer biology (Figure 1 and Table 2).

### 3.1. MicroRNAs

MicroRNAs (miRNAs) are small (~22 nt) single-stranded non-coding RNAs responsible for post-transcriptional control of gene expression. miRNAs exert their regulatory function through Watson–Crick base pairing of their 5′ region (seed sequence) with the target mRNA, generally in the 3′ UTR [41]. Regulation by miRNAs involves a plethora of biological processes, such as cell differentiation, proliferation, metabolism and apoptosis. It is therefore not surprising that dysregulation of miRNAs’ expression plays an important role in cancer biology [42]. Aberrant expression of miRNAs in cancer was first reported in 2002 when Calin et al. demonstrated the downregulation of the miRNA cluster miR-15/16 in chronic lymphocytic leukemia (CLL). The miR-15/16 cluster is located on chromosome 13q14, a region deleted in more than half of the cases of CLL and B-cell leukemia [43]. Notably, one of the miR-15/16 targets is BCL-2, which has a critical role in the malignant transformation of solid tissues, as well as lymphoid cells [44]. Following this first finding, a large number of miRNAs controlling the expression of BCL-2 family members has been identified. In 2011, a high-throughput approach integrating expression profiling and in silico prediction was employed to identify a set of miRNAs targeting BCL-2 [45]. An intriguing mechanism of BCL-2 regulation was shown in 2015 by Kuwano and colleagues. In colon cancer cells, the authors found that transformer 2β (Tra2β), a splicing factor overexpressed in many cancer tissues, modulates the accessibility of the miR-204 binding site on *BCL-2* 3′ UTR by competitive binding, thus stabilizing *BCL-2* mRNA [46].

The broad regulatory power of microRNAs relies on their ability to control whole pathways or even multiple complementary pathways by regulating the expression of different actors of the same set. Our group reported on the role of miR-197 and miR-663 in conferring apoptosis resistance in non-small cell lung (NSCL) cancer. Briefly, miR-197 behaves as a master regulator of the apoptotic response in p53 wt background by targeting NOXA and BMF [47]. MiR-663 inhibits mitochondrial outer membrane permeabilization (MOMP), downregulating PUMA and preventing p53 mitochondrial accumulation by repressing BTG2 expression [48]. Further, we showed how the miR-17–92 cluster, under the transcriptional control of N-MYC, inhibits the expression of p21 and BIM in neuroblastoma, orchestrating uncontrolled cell proliferation and apoptotic escape [49]. Another example of simultaneous regulation of multiple proteins belonging to the BCL-2 family is miR-29. During neuronal development, miR-29 expression progressively increases, and it is kept at sustained levels throughout mature neurons’ lifespan to prevent apoptosis triggering in response to different stress-inducing conditions. This pro-survival action of miR-29 ensures neuron preservation during the lifetime of the organism and is carried out by targeting different members of the pro-apoptotic BH3-only subfamily (BIM, BMF, HRK, PUMA, N-BAK) [50]. Conversely, in cancerous background, miR-29 exerts a pro-apoptotic role by multiple regulation of apoptosis effectors. Specifically, miR-29 was shown to induce apoptosis by directly targeting BCL-2 and MCL-1 and by indirectly activating p53 through the suppression of its inhibitors p85α and CDC42 [51,52,53,54]. Consistent with an oncosuppressive role, downregulation of miR-29 has been reported in multiple cancer types, where its critical role in tumorigenesis and cancer progression has been described [55].

Further, microRNAs can take part in complex regulatory circuitries, involving protein factors and other ncRNAs, where the activity of main effectors of pivotal cellular functions are finely modulated in response to intra- and extra-cellular stimuli. One striking example of such intriguing connections is the miR-34 family. MiR-34, a p53-induced microRNA, functions as a tumor suppressor by playing a role in cell cycle arrest, apoptosis and metabolic regulation [56,57,58]. miR-34 targets include the anti-apoptotic factors BCL-2, sirtuin 1 (SIRT1) and MDM4 [59]. While p53 induces the transcription of miR-34, this miRNA in turn represses MDM4 and/or SIRT1 to enhance p53 transcription activity and decrease p53 protein turnover in a positive feedback loop that boosts a robust tumor suppressive response [60]. Restoration of miR-34 expression in p53-deficient cancer cells could be exploited to restore chemosensitivity to drugs that rely on p53-mediated apoptosis, as was shown in gastric cancer cells [61].

As discussed above, pro-survival members of the BCL-2 family are involved in several hallmarks of cancer, spanning from invasion and metastasis to angiogenesis and DNA replication and repair [62]. Interestingly, miR-335 was unveiled to suppress the invasive and metastatic properties of gastric cancer cells by targeting BCL-W and SP1, and low expression of miR-335 was significantly associated with lymph-node metastasis, poor pT and pN stage, underscoring the prognostic potential of miR-335 profiling [63]. miR-335 was also found downregulated in clear cell renal carcinoma (ccRCC), and its overexpression significantly inhibits the proliferation and invasion of ccRCC cells through direct BCL-W targeting [64].

Multi-drug resistance (MDR) is one of the major dilemmas in cancer therapy, due to mutations, changes in gene expression and drug-induced karyotypic alterations. The BCL-2 family is known to have a major role in tumor evasion of drug-induced cell death [65]. MiRNAs have been explored as good candidates to overcome MDR, and a wide body of evidence shows that miRNAs can sensitize cancer cells to drug treatments. Indeed, microRNA-mediated modulation of BCL-2 family proteins proved to be an effective anti-cancer strategy in combinatorial therapies in pre-clinical models, resulting in cancer sensitization to chemotherapy. For example, miR-125b was found to sensitize hepatocellular carcinoma (HCC) cells to chemotherapeutic treatment by directly targeting MCL-1, BCL-W and indirectly targeting BCL-XL [66]. Indeed, doxorubicin-treated HCC cell lines showed a marked increase in apoptosis when miR-125-b was overexpressed, due to a downregulation of the anti-apoptotic proteins. On the contrary, inhibition of miR-125b significantly reduced drug-induced apoptosis. Another microRNA targeting MCL-1 in HCC is miR-101. Combining low doses of doxorubicin with miR-101 mimic was sufficient to significantly induce MCL-1-dependent apoptosis, thus suggesting a good strategy to overcome the toxicity of doxorubicin treatment observed in clinics [67,68,69]. Downregulation of MCL-1 by miRNAs was found to affect also the cancer stem cell (CSC) population in breast cancer. CSCs are resistant to chemotherapy and therefore are mainly responsible for treatment failure in patients. miR-519d was found to sensitize CSCs to cisplatin treatment by targeting MCL-1. MiR-519d overexpression or MCL-1 silencing alone did not affect CSCs’ viability, while the combination with cisplatin led to increased apoptosis of CSCs [70]. Furthermore, miR-34 was found to play a role in CSCs’ self-renewal and survival. MiR-34 was reported to be downregulated in the CD44+/CD133+ pancreatic CSC population, accompanied by high levels of its target gene BCL-2. Moreover, restoring miR-34 expression was shown to inhibit cell clonogenic growth and to increase cell death and response to chemo-/radio-therapy of pancreatic CSCs by the simultaneous inhibition of its downstream target genes Notch and BCL-2 [71].

Interestingly, the miR-221/222 cluster is one of the most commonly upregulated miRNAs in human solid and hematologic tumors, and it was found to target multiple BH3-only pro-apoptotic proteins. The role of the miR-221/222 cluster was studied in a model of dexamethasone-induced drug resistance in multiple myeloma (MM). By using the isogenic cell lines MM1R and MM1S, respectively resistant and sensitive to dexamethasone, the authors identified DNA copy number gains in MM1R cells that were associated with increased miR-221-222 expression and consequent downregulation of its target PUMA. Modulation of the miR-221/222 cluster alone was able to revert the resistance of MM1R cells and conversely to confer resistance to sensitive MM1S cells, thus demonstrating the key role of these microRNAs as mediators of resistance in MM. These data were consistent with observed overexpression of this miR cluster together with the decrease of PUMA in relapsed patients [72].

### 3.2. Long non-Coding RNAs

Multiple regulatory mechanisms targeting BCL-2 family members involve a different class of non-coding RNAs called long non-coding RNAs (lncRNAs).

LncRNAs have recently jumped on the non-coding RNA stage as crucial regulators of gene expression through a wide variety of molecular mechanisms. These transcripts >200 bp in length fold into evolutionarily-conserved secondary structures and exert their regulatory functions at different levels by recruiting chromatin modifiers or transcription factors on gene promoters, by affecting the assembly of protein complexes, by interfering with mRNA processing or behaving as microRNA sponges (competing endogenous RNAs (ceRNAs)) [73,74]. The identification of a plethora of lncRNAs differentially expressed in tumors relative to normal tissues or at different tumor stages hinted at their potential as diagnostic, prognostic and predictive biomarkers. Several lncRNAs dysregulated in cancer have been reported to mediate escape from apoptosis through different mechanisms.

*Lnc_ASNR* (apoptosis suppressing non-coding RNA) was identified through transcriptome profiling of different tumor specimens (gastric, colon, liver and lung) where it was found upregulated. Further analyses unveiled that *lnc_ASNR* exerted a pro-survival role in tumor cells by promoting nuclear retention of the RNA binding protein AUF1 that in physiological conditions mediates *BCL-2* mRNA degradation [75]. The finding that metastasis-associated lung adenocarcinoma transcript 1 (*MALAT1*) behaves as a ceRNA targeting tumor suppressor miR-125b unveiled a negative feedback loop in bladder cancer [76]. In fact, the same group had previously identified *MALAT1* as a target of miR-125b overexpressed in bladder cancer [77]. More recently, using CRISPR-mediated *MALAT1* activation or interference, the authors demonstrated that *MALAT1* induces miR-125b degradation through the Ago2 complex, resulting in concurrent upregulation of miR-125b target genes *BCL-2* and *MMP13*. Accordingly, silencing of BCL-2 and MMP13 neutralized the anti-apoptotic and invasion-promoting effect of miR-125b inhibitor on *MALAT1* knockdown cells, thus confirming the role of these proteins in the establishment of cancer phenotypes in a *MALAT1*-overexpressing background. Further, BCL-2 upregulation mediated by lncRNA *HOTTIP* (HOXA distal transcript antisense RNA) was found to underlie chemoresistance in small cell lung cancer (SCLC) [78]. *HOTTIP* expression was increased in human SCLC chemoresistant cell lines and tissues compared to their chemosensitive counterparts. Noteworthy, the manipulation of *HOTTIP* expression affected the drug resistance of SCLC cell lines, measured as changes in the IC_50_. In addition, the combination of *HOTTIP* knockdown and administration of cisplatin and etoposide upon subcutaneous injection in mice of chemoresistant cells showed an additive effect in reducing tumor burden. Mechanistically, *HOTTIP* acts as a competing endogenous RNA by binding miR-216a, thus abolishing the miRNA-induced downregulation of BCL-2. As described for BCL-2, lncRNAs can post-transcriptionally upregulate the anti-apoptotic relatives of the BCL-2 family by acting as miRNA decoys. In breast cancer, high expression of BCL-W showed a positive correlation with lncRNA HOX transcript antisense RNA (*HOTAIR*) levels. Mechanistically, *HOTAIR* was demonstrated to titer away miR-206, thereby relying on the *BCL-W* transcript from miRNA-induced repression [79]. A similar molecular circuitry occurred in renal cell carcinoma, where BCL-W expression was boosted owing to lncRNA *RP11-436H11.5* competitive binding to miR-335-5p [80].

LncRNA taurine upregulated 1 (*TUG1*) provides an interesting example of epigenetic regulation of *BAX* transcription. *TUG1* aberrant expression in serum samples from lung adenocarcinoma patients compared to healthy donors showed a correlation with tumor size, degree of differentiation, metastasis and TNM stage. RNA immunoprecipitation (RIP) and RNA pulldown assays in lung adenocarcinoma cells demonstrated the interaction between *TUG1* and the histone methyltransferase EZH2. Chromatin immunoprecipitation (ChIP) analysis revealed that *TUG1* mediates epigenetic silencing of *BAX* promoter through EZH2 recruitment and consequent H3K27 hypermethylation, hence conferring tumor cells’ protection against apoptotic triggers [81].

Moreover, lncRNAs proved to play a pivotal role in the activation of the p53-driven transcriptional program in response to DNA damage. By integrating RNA-seq with p53 ChIP-seq analyses in a colorectal cancer cell line under DNA damage, a signature of eighteen p53-regulated lncRNAs was identified [82]. Among them, *PR-lncRNA-1* and *PR-lncRNA-10* were required for the efficient binding of p53 to some of its target genes including *BCL2L1*, *PUMA* and *BAX*, contributing to apoptosis triggering upon DNA damage. Interestingly, some of the identified lncRNAs constituted a tumor suppressor signature with diagnostic power in colorectal cancer. Conversely, lncRNA *PANDA*, also transcribed via p53 following DNA damage, was demonstrated to hinder NF-YA-dependent transcription of *PUMA*, *NOXA* and other pro-apoptotic genes. Briefly, p53 binding at the *CDKN1A* locus coordinately activates expression of p21, as well as antisense noncoding transcript *PANDA*, which in turn promote cell cycle arrest and apoptosis blockade, respectively [83].

### 3.3. Circular RNAs

In the diverse landscape of non-coding RNAs, circular RNAs (circRNAs) represent a novel class that has gained increasing consideration. These covalently-closed circular structures arise from either exon skipping or direct back-splicing. Exon skipping leads to a lariat that later on undergoes circularization, whereas direct back-splicing refers to the coupling of a donor splice site with an upstream unspliced acceptor site, which results in the circularization of the intervening RNA. CircRNAs are highly stable in vivo compared with linear RNAs, mainly localized in the cytoplasm and can be detected in exosomes [84]. Recently, circRNAs have been shown to act as microRNA sponges to regulate gene expression [85,86]. The pro-proliferative *circHIPK3* was enriched in an RIP for Ago2, and a luciferase screening for an miRNA library identified nine growth-suppressive miRNAs that were sponged by this circular RNA, including miR-29 [52,87]. CircUBAP2, whose expression positively correlated with osteosarcoma progression, prevented apoptosis by raising BCL-2 expression through miR-143 sponging [88]. Circ-Foxo3 induces cell death through a different mechanism involving the regulation of the host gene transcript that eventually leads to PUMA overexpression. Circ-Foxo3 is downregulated in breast cancer specimens compared to the adjacent normal tissue, and nanoparticle-mediated delivery of a circ-Foxo3 plasmid in xenografts resulted in smaller tumors displaying a high percentage of TUNEL-positive cells [89]. Mechanistically, circ-Foxo3 exerts a tumor suppressor role by functioning as a scaffold for the assembly of the p53-MDM2 complex, hence releasing FOXO3 protein from MDM2-mediated ubiquitination and degradation. The resulting upregulation of the FOXO3 downstream target PUMA triggers apoptosis.

## 4. Concluding Remarks

The identification of the growing family of ncRNAs has broadened the vision of gene expression regulation, providing further layers of complexity that explain the capability of cells to rapidly adapt to external and internal stimuli [90]. Moreover, the large number of ncRNAs discovered so far provided a rationale to elucidate the contradiction between the huge extent of the human genome, 76% of which is actively transcribed as unveiled by the recent ENCODE project, and the relatively restricted number of protein-coding genes (about 20–25,000) [91,92]. The “one gene, one enzyme” paradigm stated by George Beadle in 1945 is nowadays completely reverted mainly due to ncRNAs. In this review, we focused on the role of three major classes of ncRNAs (miRNAs, lncRNAs and circRNAs) in the regulation of intrinsic apoptosis and more specifically of the BCL-2 protein family. BCL-2 family members have emerged as master regulators of the mitochondrial apoptotic machinery and potential therapeutic targets owing to their ability to induce MOMP following a wide range of stress insults, including anti-cancer drugs. Consistently, shifting the balance towards the pro-apoptotic members of the BCL-2 family may represent a powerful therapeutic strategy to overcome mechanisms of apoptosis resistance in cancer. By mimicking BH3-only proteins, BH3 mimetic drugs competitively bind pro-survival members of the BCL-2 family, thus displacing sequestered pro-apoptotic proteins [93]. The first developed compound ABT-737, targeting BCL-2, BCL-XL and BCL-W, exhibited single-agent antitumor activity in preclinical models of lymphoma and small-cell lung cancer (SCLC), as well as in primary patient-derived CLL samples [94]. Its orally-available analogue ABT-263 (Navitoclax) proved to be effective in phase I and II clinical trials in CLL, both as monotherapy [95] and in combination with standard cytotoxic chemo-immunotherapy regimens [96,97]. Unfortunately, the emergence of thrombocytopenia owing to BCL-XL targeting in platelets put a halt to its use in the clinic [98,99]. Development of the BCL-2-selective inhibitor ABT-199 (Venetoclax) circumvented this obstacle [100], and a successful phase II clinical trial in poor prognosis relapsed or refractory CLL patients led to the U.S. Food and Drug Administration (FDA) registration for Venetoclax [101]. However, innate or acquired MCL-1-dependent resistance represents the Achilles’ heel of ABT-737, Navitoclax and Venetoclax [102,103]. Among MCL-1-selective inhibitors that have been recently developed [104], AMG176 is the first compound to have reached phase I clinical evaluation (ClinicalTrials.gov identifier: NCT02675452). In addition to functional inhibitors, alternative strategies to counteract MCL-1 resistance have been explored, such as downregulation of MCL-1 expression by caloric restriction in B-cell lymphoma [105] or by cardiac glycoside UNBS1450 in AML [106]. Here, we have described several microRNAs targeting MCL-1 that have been shown to sensitize cancer cells to cytotoxicity induced by either chemotherapeutics (miR-125b, miR-101, miR-519d) or TRAIL (TNF-related apoptosis-inducing ligand) treatment (miR-29) [52]. Combining BH3 mimetics with mimics of these microRNAs may be a promising therapeutic option for cancers displaying MCL-1-mediated resistance.

Besides MCL-1-mediated resistance, modulation of ncRNAs controlling the BCL-2 family, alone or in combination with other agents, could be explored as a therapeutic strategy against cancer [107]. Unfortunately, despite the large body of pre-clinical evidence, development of ncRNA-targeted therapeutics has slowed down owing to systemic delivery issues and the emergence of toxicities. However, novel delivery strategies have been optimized, raising the hope of translating such broad regulatory potential into effective molecules in the clinical setting. EnGeneIC Delivery Vehicle (EDV) nanocells (also called TargomiRs) are bacteria-derived particles that can be modified with surface-conjugated antibodies to enable tissue-specific targeting [108]. EDV nanocells coated with epidermal growth factor receptor (EGFR)-specific antibodies entered a phase I trial for the delivery of miR-16 mimics in patients with malignant pleural mesothelioma or non-small cell lung cancer (NSCLC). The results of this first-in-man study of TargomiRs showed that a dose of 5 × 10^9^ TargomiRs per week was well tolerated and was accompanied by early signs of antitumor activity. These encouraging data support additional studies of miR-16-based TargomiRs in combination with chemotherapy or immune checkpoint inhibitors [109]. Furthermore, the application of these new frontiers of delivery to the modulation of microRNAs involved in the control of apoptosis will hopefully lead to effective therapeutic strategies to hit cancer and overcome resistance and recurrence.

## Figures and Tables

**Table 1:** STRING software was used to search for each BH3-only protein interactors. Only experimentally-validated interactions with a minimum score of 0.4 (medium confidence) are reported in the table. Interactors reported for HRK were identified by searching with a minimum score of 0.15 (low confidence).

BH3-Only Proteins	p53 Transcriptional Targets	Pro-Survival Interactors	Direct Activation of BAX/BAK
BAD	No	BCL-2, BCL-XL, BFL-1, MCL-1	-
BID	No	BCL-2, BCL-XL, BFL-1, MCL-1	√
BIK	No	BCL-2, BCL-XL, BCL-W	-
BIM	No	BCL-2, BCL-XL, BFL-1, MCL-1	√
BMF	No	BCL-2, BCL-XL, BFL-1, MCL-1	-
HRK	No	BCL-2, BCL-XL, MCL-1	-
NOXA	Yes	BCL-2, BCL-XL, BFL-1, MCL-1	-
PUMA	Yes	BCL-2, BCL-XL, BFL-1, MCL-1	-

**Table 2:** ncRNAs regulating apoptosis by BCL-2 family members’ modulation.

ncRNA	BCL-2 Family Target	Molecular Mechanism	Source
miR-15/16	BCL-2	Direct targeting	[44]
miR-204	BCL-2	Direct targeting	[46]
miR-197	NOXA; BMF	Direct targeting	[47]
miR-663	PUMA	Direct targeting	[48]
miR-17/92	BIM	Direct targeting	[49]
miR-29	BIM; HRK; BMF; PUMA; BAK; MCL-1	Direct targeting	[50,51,52,87]
miR-34	BCL-2	Direct targeting	[59,61,71]
miR-335	BCL-W	Direct targeting	[63,64,80]
miR-125b	BCL-2; BCL-W; MCL-1	Direct targeting	[66,76]
miR-125b	BCL-XL	Indirect targeting	[66]
miR-101	MCL-1	Direct targeting	[67]
miR-519d	MCL-1	Direct targeting	[70]
miR-221/222	PUMA	Direct targeting	[72]
miR-216a	BCL-2	Direct targeting	[78]
miR-206	BCL-W	Direct targeting	[79]
lnc_ASNR	BCL-2	Nuclear retention of AUF1	[75]
MALAT1	BCL-2	Competing endogenous RNA for miR-125b	[76]
HOTTIP	BCL-2	Competing endogenous RNA for miR-216a	[78]
HOTAIR	BCL-W	Competing endogenous RNA for miR-206	[79]
RP11-436H11.5	BCL-W	Competing endogenous RNA for miR-335	[80]
TUG1	BAX	Epigenetic silencing by EZH2 recruitment	[81]
PR-lncRNA-1	BAX	P53 recruitment to target genes promoters	[82]
PR-lncRNA-10	BCL2L1; PUMA; BAX	P53 recruitment to target genes promoters	[82]
PANDA	PUMA; NOXA	Inhibition of NF-YA-dependent transcription	[83]
circHIPK3	MCL-1	Competing endogenous RNA for miR-29	[87]
circUBAP2	BCL-2	Competing endogenous RNA for miR-143	[88]
circ-Foxo3	PUMA	Inhibition of MDM2-mediated FOXO3 degradation	[89]
